# Congenital cytomegalovirus infection drives oligoclonal expansion of cytotoxic γδ T cells from early fetal progenitors

**DOI:** 10.1093/jimmun/vkag099

**Published:** 2026-06-15

**Authors:** Justine Levan, Anna V Vaaben, Jessica Tsui, Brittany R Davidson, Meagan E Olive, Perri C Callaway, Catherine B T Nguyen, Mikias Ilala, Ravi K Patel, Riana D Hunter, Gabriela K Fragiadakis, Alexis J Combes, Felistas Nankya, Grant Dorsey, Abel Kakuru, Mary Muhindo, Margaret E Feeney

**Affiliations:** Department of Medicine, University of California, San Francisco, San Francisco, CA, United States; Department of Medicine, University of California, San Francisco, San Francisco, CA, United States; UCSF CoLabs, University of California, San Francisco, San Francisco, CA, United States; UCSF CoLabs, University of California, San Francisco, San Francisco, CA, United States; Department of Medicine, University of California, San Francisco, San Francisco, CA, United States; Biomedical Sciences PhD Program, University of California, San Francisco, San Francisco, CA, United States; Department of Medicine, University of California, San Francisco, San Francisco, CA, United States; Infectious Diseases and Immunity Program, University of California, Berkeley, Berkeley, CA, United States; Department of Medicine, University of California, San Francisco, San Francisco, CA, United States; Department of Medicine, University of California, San Francisco, San Francisco, CA, United States; UCSF CoLabs, University of California, San Francisco, San Francisco, CA, United States; Department of Medicine, University of California, San Francisco, San Francisco, CA, United States; Biomedical Sciences PhD Program, University of California, San Francisco, San Francisco, CA, United States; UCSF CoLabs, University of California, San Francisco, San Francisco, CA, United States; Department of Rheumatology, School of Medicine, University of California, San Francisco, San Francisco, CA, United States; UCSF CoLabs, University of California, San Francisco, San Francisco, CA, United States; Department of Pathology, School of Medicine, University of California, San Francisco, San Francisco, CA, United States; Division of Gastroenterology, Department of Medicine, University of California, San Francisco, San Francisco, CA, United States; Infectious Diseases Research Collaboration, Kampala, Uganda; Department of Medicine, University of California, San Francisco, San Francisco, CA, United States; Infectious Diseases Research Collaboration, Kampala, Uganda; Infectious Diseases Research Collaboration, Kampala, Uganda; Department of Medicine, University of California, San Francisco, San Francisco, CA, United States; Department of Pediatrics, University of California, San Francisco, San Francisco, CA, United States

**Keywords:** congenital CMV, early life immunity, gamma delta T cells

## Abstract

Gamma delta (γδ) T cells emerge early during human gestation and are uniquely equipped to protect the fetus and infant following infection, due to their innate-like recognition of conserved molecular ligands. Following congenital cytomegalovirus infection (cCMV), Vδ1 T cells have been shown to expand, differentiate, and upregulate cytotoxic mediators, but display markedly restricted γδTCR diversity compared to CMV-infected adults. Early fetal γδ T cells are biased toward rapid effector function and comprise a distinct ontological “layer” that can be distinguished from late gestation γδ T cells based on TCR characteristics. To better understand the contribution of fetal γδ T cells to antiviral defense in utero, we analyzed γδ T cells from cCMV^+^ and uninfected neonates in Uganda using flow cytometry and paired single-cell RNA and TCR sequencing. We observed that in cCMV^+^ neonates, γδ T cells clonally proliferate and differentiate into a uniform population of cytotoxic effectors. The expanded Vδ1 population is comprised of diverse clonotypes with broad Vγ chain usage, with a striking correlation between expansion and publicity. Overall, γδTCR repertoires of cCMV^+^ infants have shorter CDR3 lengths and fewer N additions, suggesting they derive from early fetal progenitor cells. In cCMV^+^ infants, most cells with fetal γδTCR characteristics are effector-differentiated, whereas in uninfected infants, such cells are rare and predominantly naïve. Together, these findings demonstrate that cCMV infection drives an oligoclonal expansion of highly cytotoxic effector γδ T cells with fetal-like TCR features, illustrating how the developing immune system prioritizes broad reactivity and rapid effector function over specificity.

## Introduction

The human fetus and young infant are uniquely susceptible to infections, as they lack adaptive immune memory from prior encounters with pathogens. Thus, to counter infectious threats, the fetus relies on immune cell populations and differentiation programs that are poised for rapid effector function.^1^ T cells expressing the γδTCR are the first to develop during human gestation, appearing in the fetal liver as early as 6 to 9 weeks.^2^ They recognize non-peptide antigens and possess many features that make them uniquely suited to protecting the fetus and infant, including rapid, innate-like effector functions that are not dependent on prior antigen exposure or priming by dendritic cells, which are functionally immature in early life.^3–7^ Further, they are MHC-unrestricted and exhibit remarkable functional plasticity, including cytolytic, antibody-mediated, and antigen-presenting functions.^8–11^ In some infection models, γδ T cells are required for the protection of young, but not mature, mice.^12^ Indeed, it has been hypothesized that the primary selective advantage driving the γδTCRs over 450 million years of conservation as a third class of V(D)J-recombined antigen receptors is its role in neonatal protection.^13^

Human cytomegalovirus (CMV) is the most common congenital infection worldwide.^14^ It is a leading cause of neurodevelopmental delays in newborns, and the clinical consequences of in utero infection may be severe, including microcephaly, hepatosplenomegaly, jaundice, chorioretinitis, hearing loss, or being small for gestational age.^15^^,^^16^ In adults, CMV infection induces an expansion of γδ T cells that can lyse CMV-infected target cells in vitro.^17–22^ The precise ligand(s) targeted by γδ T cells in CMV-infected individuals are largely unknown and may include viral-encoded or stress-induced host cell ligands.^23–26^ While specific subsets of γδ T cells have also been observed to expand in congenitally CMV-infected (cCMV) infants, the γδTCR repertoire in such infants is notably restricted, oligoclonal, and highly shared. One study of European infants demonstrated the presence of a highly shared (“public”) Vγ8^+^Vδ1^+^ clonotype that was entirely germline-encoded and present in nearly all CMV-infected neonates.^27^ In contrast, CMV^+^ adults possess diverse and largely private repertoires.^22^^,^^28^^,^^29^ The observed differences between the adult and fetal γδTCR cell repertoires upon infection with CMV may be explained by the “layered” model of immune development, which posits that the immune system develops via successive waves of T cells that arise from distinct progenitor populations differing in their intrinsic properties and developmental potential.^1^^,^^30–32^ During murine development, γδ T cells emerge in distinct waves that are distinguishable by their Vγ and Vδ chain usage; these subsets arise sequentially and populate the periphery to assume functionally specialized roles.^33^^,^^34^ Recent data indicate that human γδ T cells also emerge in discrete waves that derive from distinct hematopoietic precursor populations and exhibit differences in antigen recognition, functional programming, and other intrinsic properties.^32^^,^^35–38^ In humans, these developmental waves are not as easily defined by Vδ or Vγ chain usage, yet detailed analyses of γδTCRs of mid-gestation human thymocytes have revealed distinct sequence characteristics associated with early fetal origin, including limited junctional diversity and preferential gene segment usage.^35^ Importantly, these mid-gestation γδ T cells exhibit effector-biased transcriptional programming, even prior to thymic egress or encounter with pathogens.^35^

Given their unique significance in early-life immunity, we sought to determine how successive waves of γδ T cells in the developing fetus respond to cCMV infection. Here, we employed flow cytometry and paired single-cell transcriptional and TCR sequencing (TCR-seq) profiling of individual cord blood γδ T cells from cCMV-infected and uninfected Ugandan infants to examine how in utero infection with CMV influences the phenotype, gene expression, TCR repertoire, and effector functions of γδ T cells in the fetus. In addition, we leveraged the distinct γδTCR features that distinguish early fetal progenitor populations to probe the contribution of these early-rising cells to the immune response of cCMV-infected newborns. Our findings suggest that cCMV infection triggers an oligoclonal expansion of γδ T cells that adopt a remarkably uniform effector phenotype characterized by high expression of cytotoxic granule–associated molecules and the persistence of a distinct fetal transcriptional signature.

## Materials and methods

### Study populations and samples

Cord blood mononuclear cells (CBMCs) were obtained from a subset of infants (*n* = 85) enrolled in a clinical trial of prenatal and early childhood malaria chemoprevention conducted in the Tororo and Busia Districts, a highly malaria-endemic area in Eastern Uganda (PROMOTE-BC1 [NCT02163447]; PROMOTE-BC3 [NCT02793622]). Details of the larger study, including eligibility criteria for enrollment and clinical outcomes, have been previously described in detail.^39^ All pregnant women in this trial screened negative for human immunodeficiency virus (HIV) at enrollment. Umbilical cord blood was collected at delivery using cord blood collection kits (Pall Medical) and an aliquot of whole cord blood preserved in RNAlater (Thermo Fisher) at −80 °C. CBMCs were promptly isolated from the remaining cord blood using density gradient centrifugation (Ficoll-Histopaque; GE Life Sciences) and cryopreserved in liquid nitrogen.

### Ethical approval

Written informed consent was obtained from the mothers of all study participants. The study protocol was approved by Makerere University School of Biomedical Sciences Ethics Committee, Uganda National Council of Science and Technology, and the University of California, San Francisco (UCSF) Research Ethics Committee.

### Congenital CMV screening

To identify cCMV-infected newborns, DNA was extracted from whole cord blood preserved in RNAlater using the QIAamp DNA Blood Mini Kit (Qiagen) according to the manufacturer’s instructions. Extracted DNA was then quantified with a Qubit 4 Fluorometer (Invitrogen) before screening for CMV viral DNA by quantitative real-time PCR (qRT-PCR) using PowerUp SYBR Green Master Mix (Applied Biosystems) on the ABI StepOne Plus system. All samples were screened in duplicate using primers targeting UL123 (Table S1).^40^ CMV-positive samples were retested with a second qRT-PCR using a UL55 primer set to confirm positivity (Table S1).

All discordant qRT-PCR results were rescreened by droplet digital PCR (ddPCR) as published previously.^41^ In brief, 5 μL of extracted DNA was added to 10 μL of ddPCR 2X PCR master mix (Quantalife, Applied Biosystems) in addition to 1 μL each of 10 μM forward and reverse primers and 1 μL of 5 μM probe targeting UL55; the same primers were used for qRT-PCR and ddPCR (Table S1). The combined DNA and master mix (18 μL) was added to the droplet generator strips and placed in the automated droplet generator, and the resulting emulsion was transferred to a 96-well PCR plate. After amplification, the number of positive and negative droplets were enumerated using a Bio-Rad QX300 droplet reader based on fluorescence.

### Flow cytometry

CBMCs were thawed, counted, evaluated for viability, and stained for extra- and intracellular targets with antibodies listed in Table S2. CBMCs were stained with LIVE/DEAD Fixable Near-IR (Thermo Fisher) to exclude dead cells. For intracellular staining, CBMCs were fixed using a Cytofix/Cytoperm kit (BD) and stained in Permeabilization/Wash buffer (BD) per manufacturer’s instructions. Data were acquired on an LSR II (BD) flow cytometer using FACS DIVA software. Compensation was performed using single-color stained UltraComp beads (Invitrogen) and a minimum of 500,000 events were recorded from each sample. Flow cytometry data were analyzed using FlowJo version 10.10.0 (Tree Star). Fluorescence minus one (FMO) controls were used to set gates for all markers. γδ T cells were defined as LIVE/DEAD-negative single cells that were CD14^−^/CD19^−^/CD3^+^ and positive for at least one of the γδTCR chains (Vδ1, Vδ2, or Vγ9) (Fig. S1A).

### Single-cell RNA and TCR sequencing

#### Library generation and sequencing

For single-cell RNA sequencing (scRNA-seq) and single-cell TCR sequencing (scTCR-seq), 10,000 Vδ1^+^ T cells were FACS sorted from cryopreserved CBMCs from 4 cCMV^−^ neonates and 6 cCMV^+^ neonates. Gene expression libraries were constructed using the Chromium Next GEM Single Cell 5′ Library & Gel Bead Kit v1.1 (10x Genomics). TCR libraries were constructed using the Chromium Single Cell V(D)J Enrichment Kit, Human T Cell v1.1 (10x Genomics). Manufacturer’s protocols for library construction were followed, except for the use of custom primers targeting the delta chain of the TCR (TCRδ) and the gamma chain (TCRγ) for TCR enrichment, published previously (Table S1).^42^ Throughout library construction, Agilent Bioanalyzer High Sensitivity DNA chips and a Bioanalyzer 2100 machine were used to check cDNA and library quality (Agilent Technologies). scRNA-seq libraries were pooled for a targeted read depth of 20,000 reads/cell pooled for a targeted read depth of 5,000 reads/cell. Pooled libraries were sequenced on the Illumina NovaSeq6000 platform.

#### Single-cell RNA gene expression demultiplexing, doublet removal, and normalization

After sequencing, Cell Ranger v7.1.0 with the default parameters was used to align the scRNA-seq libraries to the human reference genome GRCh38 (University of California, Santa Cruz [UCSC]), filter and count barcodes and unique molecular identifiers (UMIs), and generate expression matrices. The pooled libraries comprised 9 participants and were demultiplexed by using single-nucleotide polymorphism (SNP) profiles to match individual cells to the original sample. First, pileup files were generated to summarize the reads at alleles of common SNP sites derived from the 1000 Genomes Consortium.^43^ Next, Freemuxlet was used to cluster cells on similar SNP profiles and assign droplet type (singlet, doublet, or ambiguous).^44^^,^^45^ These Freemuxlet clusters were then mapped to previously generated bulk RNA-seq genotypes of the participants using BCFtools v1.10.2. Barcodes designated as singlets were then carried forward into further processing with DoubletFinder to identify and remove heterotypic intra-sample doublets.^46^ For DoubletFinder, the data were transformed using Seurat v5.2.1’s SCTransform v2; principal component analysis (PCA) was performed using npcs = 35, nearest neighbors computed via FindNeighbors(), and the data clustered using FindClusters(). DoubletFinder’s paramSweep_v3() was used to estimate the neighborhood size (pK) per library. An effective heterotypic doublet rate was estimated using the doublet proportion calculated by Freemuxlet divided by (number of samples − 1) to represent all possible intersample doublets. Further specific details on DoubletFinder parameters and implementation are published.^47^

#### Single-cell RNA gene expression integration, filtering, dimensionality reduction, and clustering

Following demultiplexing and singlet identification, Seurat v5.2.1 was used to integrate the normalized data using the canonical correlation analysis method built into the IntegrateData() function. For this, we selected the first 3,000 variable features to identify integration anchors in Seurat’s FindIntegrationAnchors() function and then integrated the normalized data using default inputs. Following integration, low-quality cells were filtered out of the resulting Seurat object. A cell meeting any of the following criteria was removed: <250 or >2,000 detected genes, ≥10% mitochondrial gene percentage, or >1% of reads mapping to hemoglobin genes. Mitochondrial and ribosomal percentages, cell cycle state, and percent of reads mapping to TCR genes were regressed out of the integrated, filtered Seurat object using SCTransform with vst.flavor = “v2.”

For clustering, we performed PCA and selected the first 40 principal components as the input for dimensionality reduction using uniform manifold approximation and projection (UMAP) with n.neighbors = 20, min.dist = 0.5 (all other parameters were default). The shared nearest-neighbor graph was then constructed using the same principal components and this graph subsequently used to identify cell clusters via Seurat’s FindClusters() k-nearest neighbor clustering with the Louvain algorithm. Clustering was performed at a series of resolutions, and the package clustree v0.5.1^48^ was used to visualize how cells move as the number of clusters increases, thereby aiding in selecting an appropriate resolution (resolution = 0.2). Marker genes for each cluster were identified using FindAllMarkers() and Wilcoxon rank-sum test method with a logfc.threshold = 0.264 (average fold change of at least 1.2), min.pct = 0.05, and an adjusted *P* value of at least 0.05.

#### Module scoring

Scores for gene set modules were calculated using Seurat’s AddModuleScore() function. Naïve, Vδ1 cytotoxic lymphocyte (CTL), and type 3 Immunity gene sets derive from published work.^49^ All genes associated with the Gene Ontology (GO) terms “Positive Regulation of T Cell Proliferation” (GO:0042102) or “T Cell Proliferation” (GO:0042098) were downloaded from the Molecular Signatures Database (MSigDB).^50^^,^^51^ See Table S3 for full gene sets.

### Single-cell TCR expression data analysis

#### γδTCR annotation and clonotype calling

The scTCR-seq libraries were aligned to the GRCh38 reference genome (UCSC) using Cell Ranger vdj v7.1.0 and v3.0.2. Because Cell Ranger vdj versions 3.1 or greater correctly align γδTCR contigs but do not accurately annotate them, the aligned output from Cell Ranger vdj v.7.1.0 was processed via the Python package Dandelion v0.5.2 for reannotation.^52^ In brief, the FASTA and contig annotation files resulting from Cell Ranger vdj were put through the standard Dandelion pre-processing, which annotates the contigs according to the latest ImMunoGeneTics (IMGT) human reference libraries and outputs Adaptive Immune Receptor Repertoire-formatted calls. As a secondary method, scTCR-seq libraries were also aligned to the genome using Cell Ranger v3.0.2. From these, we only kept the contig calls for the set of cells that did not already have γδTCR sequences called by v7.1.0 and Dandelion.

To determine TCR clonotypes, we used the Dandelion-annotated sequences as input for Scirpy v0.19.0, first using scirpy.pp.ir_dist() to compute distances between CDR3 nucleotide or amino acid (aa) sequences. We then defined clonotypes with identical CDR3 sequences at the nucleotide level using scirpy.tl.define_clonotypes() with default parameters and dual_ir = “primary”. We defined clonotypes with identical CDR3 sequences at the amino acid level using scirpy.tl.define_clonotype_clusters() with parameters dual_ir = “primary,” metric = “identity,” and sequence = “aa.” The clonotype calls, gene segment calls, CDR3 sequences, and junction sequences output from Scirpy were then incorporated into the Seurat object as metadata based on cellular barcodes.

#### Calculating N additions

The web-based Junctional Analysis Tool from IMGT was used to calculate N additions (non-templated nucleotides added at TCR gene junctions). For each cell, the V and J gene names and alleles, plus the junction nucleotide sequence, were put into FASTA format and then submitted via the website for analysis. All additional options were left as the defaults. The total number of nucleotides at all N regions was summed to yield the final number of N additions, and the delta and the gamma chains were analyzed separately.

#### scRepertoire, clonal frequency, and publicity

scRepertoire v2.2.1^53^ was used for clonal and repertoire analysis. The clonotype information from scirpy was reformatted to fit scRepertoire’s expected data structure, and the Seurat object split based on participant ID. scRepertoire’s combineExpression() function was subsequently employed to calculate the clonal frequency and proportion for each clonotype within an individual’s TCR repertoire, and then that information was incorporated back into the Seurat object as metadata based on matching cellular barcodes. For the above, a clonotype was defined using the amino acid sequences of either the gamma or delta CDR3. In some cells where sequencing failed to capture information for both chains, the missing chain information was imputed using cells from the same individual that possessed an identical gamma or delta CDR3 and that did have both TCR chains sequenced.

Publicity was defined for the TCRγ and TCRδ chains separately, by comparing the CDR3 amino acid sequence to all other existing CDR3 sequences across all individuals for the respective chain. A CDR3 sequence was considered private if it appeared only in one individual and public if it appeared in ≥2 individuals, with an additional calculation for how many other individuals it appeared in.

#### Differential gene expression analysis

For differential gene expression analysis between fetal-derived versus postnatal γδ T cells, we used the model-based analysis of single cell transcriptomics (MAST) algorithm (https://github.com/RGLab/MAST/)^54^ with cluster identity and cCMV status as latent variables and modified to use participant as a random-effect variable to account for autocorrelation of per cell gene expression within a subject (https://github.com/CBMR-Single-Cell-Omics-Platform/SCOPfunctions/blob/main/R/differential_expression.R). MAST was used to determine genes and *P* values while the default calculation from Seurat was used to determine average log fold change.

For network plots, the upregulated differentially expressed genes (DEGs) or downregulated DEGs derived via MAST above were used as the gene list for GO enrichment in clusterProfiler v.4.14.4. The resulting enriched GO terms and associated genes were then plotted using the cnetplot() function from the package enrichplot to create network plots.

### Data visualization

The R packages ggplot2,^55^ dittoSeq,^56^ ggridges,^57^ and colorspace^58^ were used to plot the flow cytometry and scRNA-seq and TCR-seq data. Treemap plots were made using the package treemapify.^59^

### Statistical analysis

Statistical testing for all plots was done in R v4.4.2 using the package ggpubr v0.6.0.^60^ Two-group comparisons were performed using an unpaired Wilcoxon rank-sum test; *P* values <0.05 were considered significant. Multiple-group comparisons were conducted using Kruskal–Wallis tests, with Dunn post hoc analysis applied to evaluate pairwise differences. Two-group comparisons of cumulative frequency distributions were performed using the Kolmogorov–Smirnov test; *P* values <0.05 were considered significant.

## Results

### CMV infection in utero results in expansion, effector differentiation, and proliferation of multiple γδ T-cell subsets

**Figure 1.**
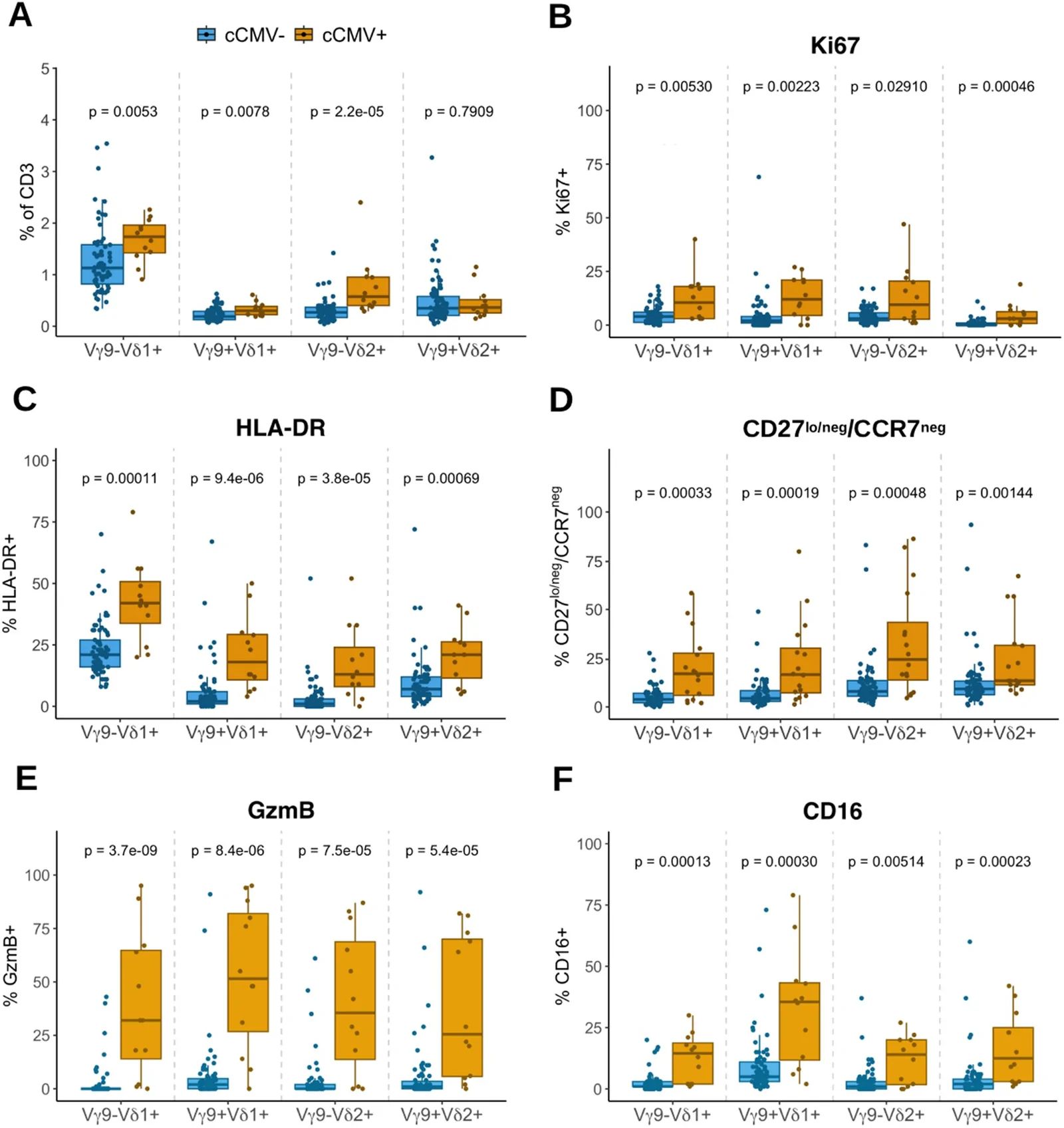
γδ T cells expand and differentiate in response to cCMV. (A) Quantification of γδ T-cell subsets in cord blood of cCMV^−^ (blue; *n* = 73) or cCMV^+^ (orange; *n* = 12). Frequency of Ki67^+^ (B), HLA-DR^+^ (C), CD27^lo/neg^/CCR7^neg^ (D), granzyme B (E), or CD16^+^ (F) cells in Vγ9^−^Vδ1^+^, Vγ9^+^Vδ1^+^, Vγ9^−^Vδ2^+^, or Vγ9^+^Vδ2^+^ γδ T-cell subsets in cCMV^−^ or cCMV^+^ infants. All flow plots were gated on live CD14^−^CD19^−^CD3^+^ T cells. *P* values were determined by Wilcoxon rank-sum test.

To characterize the γδ T-cell response to cCMV infection, we performed flow cytometric analysis on CBMCs collected from 12 cCMV^+^ and 73 cCMV^−^ neonates from Eastern Uganda. We labeled γδ T cells using antibodies recognizing the Vδ1, Vδ2, and Vγ9 TCR chains and evaluated the frequency of 4 major subsets: Vγ9^−^Vδ1^+^, Vγ9^+^Vδ1^+^, Vγ9^−^Vδ2^+^, and Vγ9^+^Vδ2^+^. Among both cCMV-infected and cCMV-uninfected neonates, Vγ9^−^Vδ1^+^ cells represented the largest population of γδ T cells analyzed, comprising from 0.34% to >3% of all T cells (Fig. 1A). Neonates with cCMV infection had significantly higher frequencies of Vγ9^−^Vδ1^+^, Vγ9^+^Vδ1^+^, and Vγ9^−^Vδ2^+^ γδ T cells compared to uninfected neonates, but similar frequencies of Vγ9^+^Vδ2^+^ cells (Fig. 1A). These data align with previous reports that both Vδ1^+^ and Vγ9^−^Vδ2^+^ T cells expand in response to CMV infection in adult solid organ transplant recipients,^18^^,^^19^ as well as with more limited data from congenitally infected infants.^27^ Interestingly, staining with Ki67 indicated that proliferation of all 4 T-cell subsets was higher in cCMV^+^ infants, including the Vγ9^+^Vδ2^+^ cells, which were not increased in frequency (Fig. 1B). This suggests that while only particular γδ T subsets expand in response to cCMV infection, the infection may create an immune milieu that promotes in vivo proliferation of all γδ T cells.

We next investigated whether these higher frequencies of γδ T-cell subsets were accompanied by changes in activation and differentiation. We found that cCMV-infected neonates had a higher frequency of activated, HLA-DR^+^ cells in all γδ T subsets analyzed (Fig. 1C). We then assessed memory differentiation by staining for CD27 and CCR7. Previous publications have defined γδ T-cell differentiation based on CD27 expression, with high CD27 expression being associated with naïve cells and low or negative CD27 expression being associated with differentiated cells that are more responsive to short-term stimulation.^61^ CD27/CCR7 co-expression is observed on naïve cells present at high frequencies in early life, whereas CCR7^neg^ γδ T cells produce more cytokines.^49^^,^^62^ Using this framework (Fig. S1B), we found that across all 4 γδ T subsets, cCMV^+^ neonates had significantly greater proportions of CD27^lo/neg^CCR7^neg^ cells, suggesting greater differentiation (Fig. 1D). Additionally, within these differentiated CD27^lo/neg^CCR7^neg^ cells, cCMV-infected neonates exhibited a more dramatic loss of CCR7, indicating a shift in the trafficking of these cells toward peripheral tissues rather than recirculation back to the lymph node.

To further understand the functional differentiation of γδ T cells in response to in utero CMV infection, we measured expression of cytotoxic molecules granzyme B (GzmB) and perforin, as well as the low-affinity IgG-Fc receptor CD16. We observed dramatically upregulated expression of GzmB in cCMV^+^ neonates across all γδ T-cell subsets (Fig. 1E). GzmB was rarely expressed by γδ T cells from uninfected neonates, whereas in cCMV^+^ neonates, expression was seen in up to 95% of cells. These GzmB^+^ cells also displayed upregulated perforin expression in cCMV^+^ infants (Fig. S2). In addition, cCMV-infected neonates displayed significant upregulation of CD16 (Fig. 1F), which could enable TCR-independent killing of antibody-opsonized CMV-infected cells.^9^

We also explored whether clinical and demographic parameters influenced the frequency or phenotype of the observed γδ T-cell subset expansions (Table S4). The frequencies of Vδ1^+^ and Vγ9^−^Vδ2^+^ cells did not differ by sex, nor did expression of activation, differentiation, or cytotoxicity markers. These parameters also did not differ significantly between cCMV^+^ infants who were small for gestational age or microcephalic at birth and those who were not. Because infants in this cohort were born in a region of high malaria endemicity, we also assessed associations with histopathologic evidence of placental malaria. However, we found no difference in γδ T subset frequency, expression of proliferation or activation markers, or CD16 between placental malaria-exposed and unexposed infants.

In sum, profiling of cord blood γδ T cells from cCMV-infected and uninfected neonates indicates that Vδ1^+^ and Vγ9^−^Vδ2^+^ T cells expand and undergo a high degree of cytotoxic effector differentiation in response to CMV infection in utero, and reveals that γδ T cells from cCMV-infected neonates—regardless of subset—exhibit a greater degree of proliferation, activation, and memory differentiation.

### Transcriptional profiling reveals marked cytotoxic effector differentiation of Vδ1^+^ T cells in cCMV infection

Next, we sought a deeper, unbiased probing of how γδ T cells alter their transcriptional programming in response to cCMV infection. For this analysis, we focused on Vδ1^+^ T cells, which were the most abundant γδ T-cell subset in the cord blood of most infants, particularly those with cCMV. We sorted Vδ1^+^ T cells (irrespective of γ chain) from the cord blood of 6 cCMV^+^ and 4 cCMV^−^ neonates and processed them for parallel single-cell RNA-seq and TCR-seq to investigate the impact of cCMV infection on Vδ1^+^ T-cell gene expression programs and TCR repertoires.

**Figure 2.**
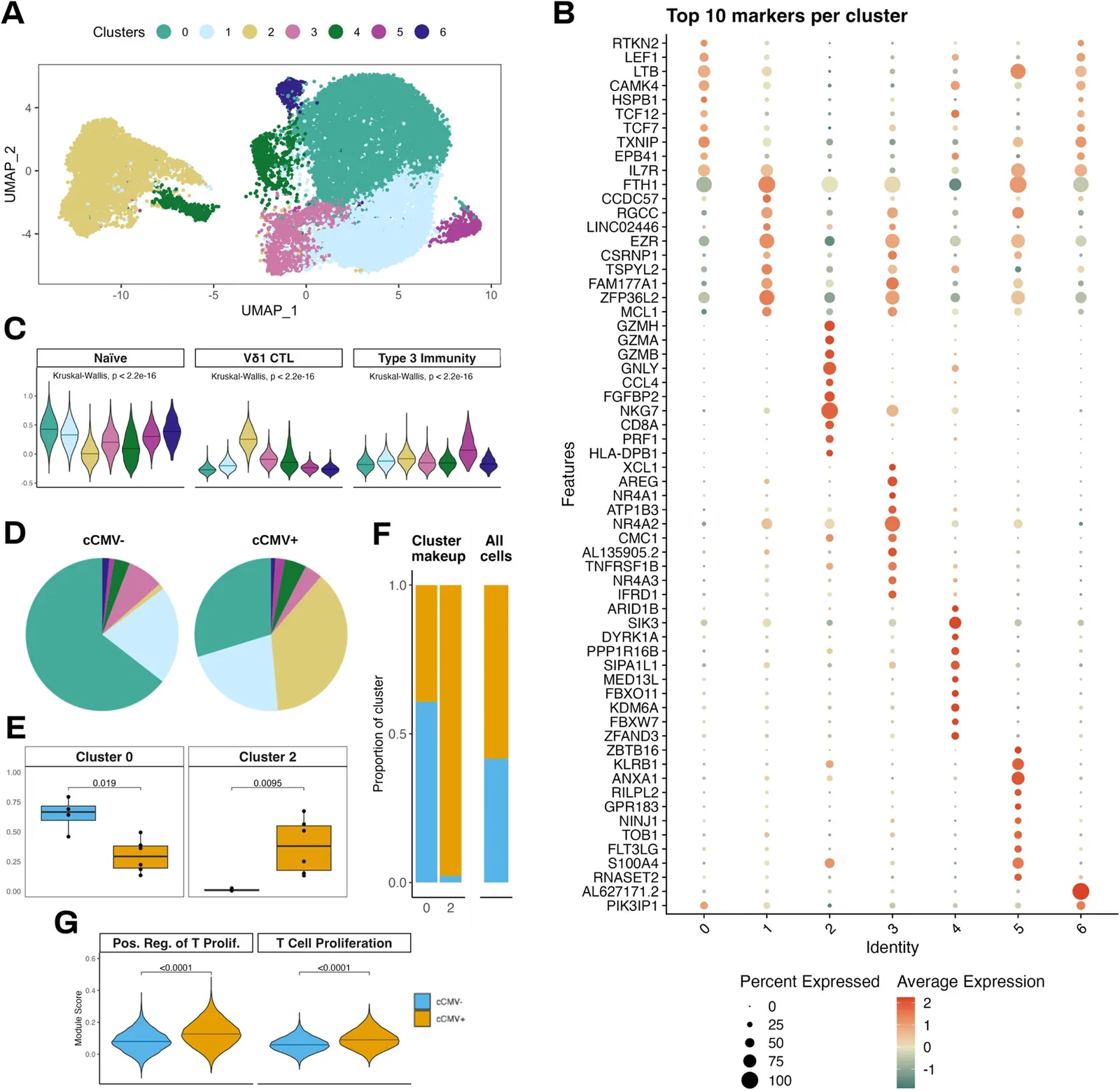
Vδ1^+^ T cells from cCMV^+^ neonates exhibit highly cytotoxic effector programming and greater proliferation. (A) UMAP projection of sorted Vδ1^+^ γδ T cells from cord blood of cCMV^−^ (*n* = 4) or cCMV^+^ newborns (*n* = 6). (B) Dotplot of the top 10 gene markers per cluster. (C) Violin plot of module score per cluster for naïve gene set, cytotoxic Vδ1 gene set, or type 3 immunity gene set. Horizontal bars indicate median scores for all cells in the cluster. *P* values were determined by Kruskal–Wallis test. (D) Pie graphs displaying the cluster distribution for cells from cCMV^−^ individuals (*n* = 11,203) or all cCMV^+^ individuals (*n* = 15,709). (E) Boxplot displaying frequency of cells that appear in cluster 0 or cluster 2 per individual. Medians and IQR for cCMV^−^ (blue) or cCMV^+^ (orange) neonates shown. (F) Stacked barplots showing the makeups of cluster 0 or cluster 2 (the proportion that is cCMV^−^ vs cCMV^+^ cells), left, and the makeup of the entire object, right. (G) Violin plot of module scores for cell proliferation gene sets from MSigDB for cCMV^−^ cells (blue) or cCMV^+^ cells (orange); Positive Regulation of T Cell Proliferation gene set (GO:0042102), T Cell Proliferation (GO:0042098). For (*E*) and (G), *P* values were determined by Wilcoxon rank-sum test.

After processing, sequencing, and quality control, we recovered gene expression data from 11,203 total Vδ1^+^ T cells from cCMV^−^ individuals and 15,709 total cells from cCMV^+^ individuals for analysis (average gene number = 675; average UMI number =1,021). Dimensionality reduction via UMAP and Seurat’s unsupervised Louvain clustering identified 7 major clusters among Vδ1^+^ T cells (Fig. 2A). The top 10 differentially expressed gene markers for each cluster demonstrated their diverse expression profiles (Fig. 2B). Cluster markers stratified by cCMV status were largely similar to those identified using all cells (Fig. S3). The largest cluster (cluster 0, containing 29.4% of the total cells) was marked by high expression of genes indicative of naïve T cells and factors associated with stemness, such as *TCF7*, *LEF1*, *IL7R*, and *LTB.*^63^^,^^64^ Cluster 6 broadly shared this transcriptomic profile, with the addition of high *PIK3IP1* expression, a negative regulator of the PI3K-Akt signaling pathway. In contrast, cells in cluster 2 exhibited highly cytotoxic type I programming, characterized by the upregulation of multiple granzymes (*GZMH*, *GZMB*, *GZMA*), granulysin (*GNLY*), perforin (*PRF1*), and other CTL-associated genes (*NKG7*, *CD8A*). Cluster 3 had high expression of amphiregulin, a hallmark wound healing cytokine produced by multiple cell types,^65–67^ in addition to transcription factors associated with thymic development (*NR4A1*, *NR4A3*).^68^ The differentially expressed markers for cluster 5 contained genes related to innate-like and type 3 signatures, such as *S100A4*, *S100A6*, *KLRB1* (which encodes CD161), and *ZBTB16* (which encodes the transcription factor PLZF).^49^ To further substantiate the functional identities of these clusters, we scored the clusters using γδ T-cell gene modules derived from the literature (Table S3).^49^ Using the built-in Seurat function FindModuleScore, the average expression levels for 3 gene sets—naïve γδ T, Vδ1^+^ CTL, and type 3 immunity—were calculated for each cell. The distribution and median of scores for each cluster (Fig. 2C) supported our annotations based on top DEGs: Clusters 0 and 6 scored the highest out of all the clusters for the naïve gene set, whereas cluster 2 scored both highest for the Vδ1 CTL set and lowest for the naïve set, and cluster 5 scored the highest for type 3 immunity. Thus, gene expression profiling indicates that cord blood Vδ1^+^ T cells are transcriptionally and functionally diverse.

Next, we assessed how the distribution of Vδ1^+^ T cells among these clusters differed between cCMV-infected and uninfected neonates (Fig. 2D). We found that infants infected in utero with CMV had a dramatically larger proportion of cells in the cytotoxic cluster 2 and substantially fewer cells in the naive cluster 0 compared to uninfected neonates (Fig. 2D). This lower proportion of naïve cells in cCMV^+^ infants corroborates our observations by flow cytometry (Fig. 1D). These differences were consistently observed across individuals, with a significantly lower frequency of naïve cluster 0 cells and a significantly higher frequency of cytotoxic cluster 2 cells among cCMV^+^ infants (Fig. 2E). Strikingly, nearly all cells in cluster 2 (97.8%) originated from cCMV^+^ infants (Fig. 2F), despite the roughly equal total cell numbers between groups overall. Cells in clusters 4 and 5 were also disproportionately derived from cCMV^+^ infants (Fig. S4A); however, the overall frequencies of cells in these clusters were lower and more variable (Fig. S4B). The naïve cluster 0, in contrast, had a greater proportion of Vδ1^+^ cells derived from cCMV^−^ infants (Fig. 2F), as did clusters 3 and 6 (Fig. S4A).

Finally, having observed by flow cytometry that γδ T cells of all subsets were more proliferative in cCMV^+^ infants (Fig. 1B), we were interested in whether γδ T cells from cCMV^+^ infants generally possessed a more broadly proliferative transcriptional program compared to cCMV^−^ infants. We therefore scored all cells using the gene sets that comprise the GO terms “Positive Regulation of T Cell Proliferation” and “T Cell Proliferation” from MSigDB.^51^ Cells from cCMV^+^ neonates scored higher for both proliferation-associated gene modules than cells from cCMV^−^ neonates (Fig. 2G). These findings together suggest that highly cytotoxic Vδ1^+^ T-cell effectors proliferate and expand to mediate an antiviral response to CMV in utero.

### Vδ1^+^ γδTCR repertoires of cCMV^+^ infants display oligoclonal expansions

**Figure 3.**
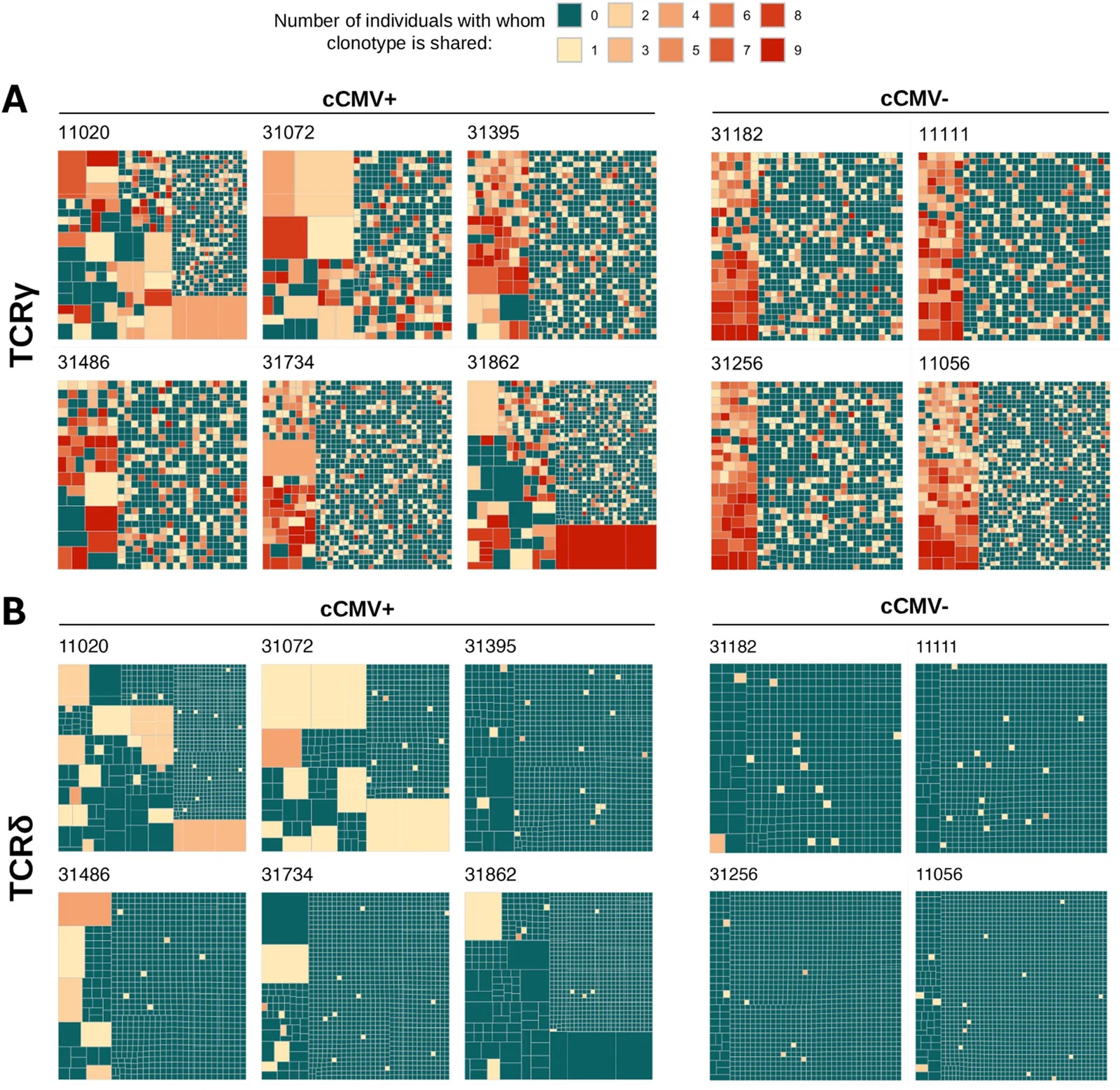
Tree maps of Vδ1^+^ TCR repertoires from cCMV^+^ and cCMV^−^ neonates. (A) Tree map representations of the TCRγ repertoires of cCMV^+^ (*n* = 6) or cCMV^−^ (*n* = 4) individuals. (B) Tree map representations of the TCRδ repertoires of cCMV^+^ (*n* = 6) or cCMV^−^ (*n* = 4) individuals. For (A) and (B), color scale indicates how many other individuals the TCR clonotype is shared with; each rectangle within a repertoire represents one clonotype and size of the rectangle corresponds to the proportion of the total repertoire that the clonotype occupies.

We additionally sequenced γδTCRs from these same Vδ1^+^ cells to determine how cCMV infection alters their diversity and clonal composition and to explore their developmental origin. We recovered TCR sequences for 18,619 cells (69.5% of total cells), identifying a TCRδ in 79% of cells and a TCRγ in 74.7%. To visualize the diversity of the TCRγ and TCRδ repertoires within each individual and the degree of clonotype sharing across individuals, we generated treemap plots, with each TCR clonotype colored by the number of other individuals also possessing that clonotype (Fig. 3). These plots illustrate that all cCMV^+^ neonates exhibited oligoclonal expansions in both their TCRγ and TCRδ repertoires, with some clonotypes comprising up to 14% of total Vδ1^+^ cells in the infant’s repertoire. Such oligoclonal expansions were not observed in uninfected neonates.

**Figure 4.**
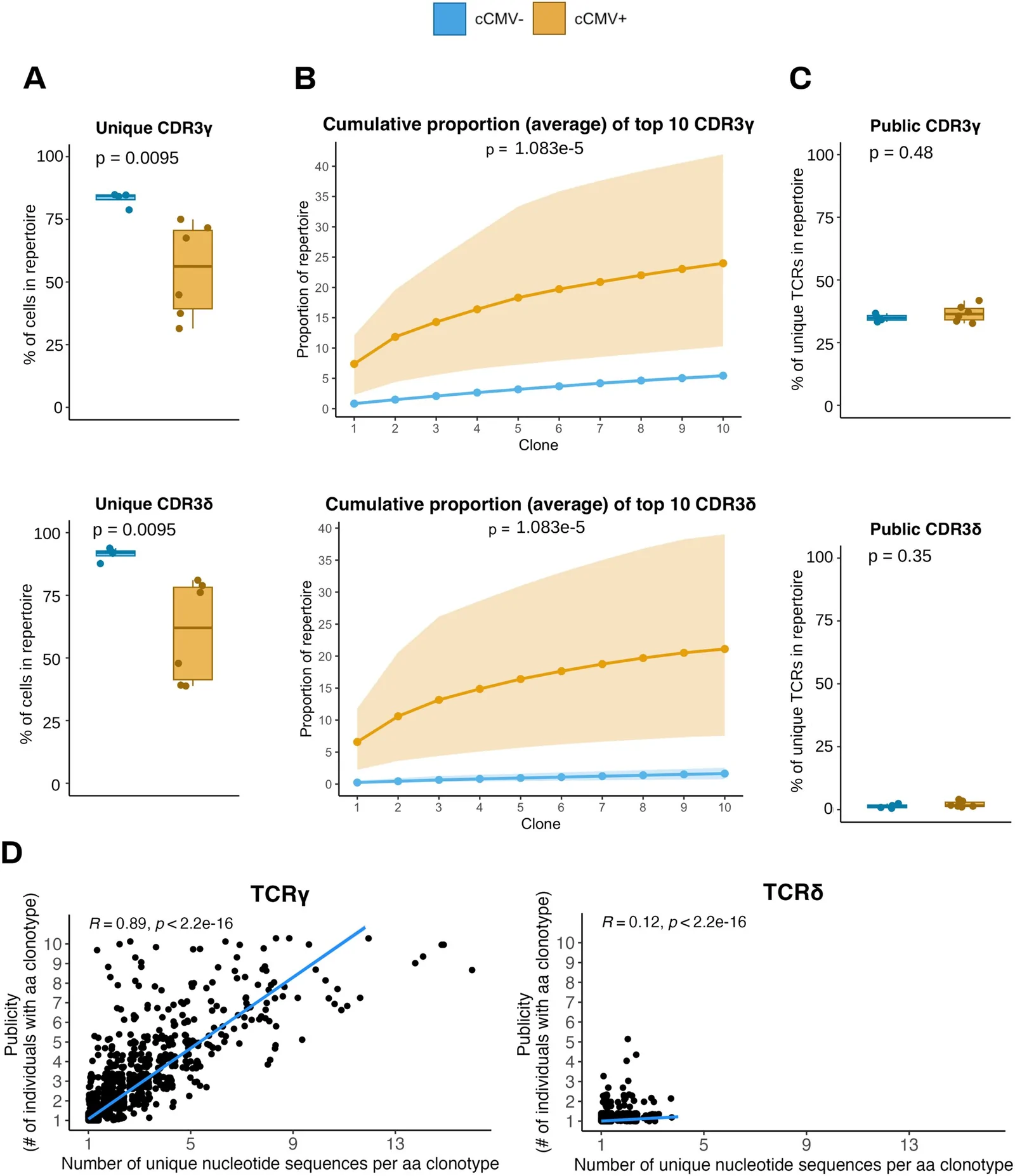
Vδ1^+^ TCR repertoires of cCMV^+^ infants are more oligoclonal than those of cCMV^−^ infants and possess clonotypic expansions. (A) Percentage of total cells per an individual’s repertoire that have a TCRγ (top) or TCRδ (bottom) that appears on only one cell, for cCMV^−^ (blue) or cCMV^+^ (orange). Each point is an individual subject. Total number of unique CDR3δ sequences = 10,215; total number of unique CDR3γ sequences = 6,586. (B) Accumulation frequency plots representing the cumulative percentage of the repertoire for the top 10 most prevalent TCRγ (top) or TCRδ (bottom) clonotypes in cCMV^−^ individuals (blue) or cCMV^+^ individuals (orange). Points and solid line indicate mean for the group while shaded area indicates the minimum and maximum values. *P* value was determined by Kolmogorov–Smirnov 2-sample test. (C) Percentage of distinct TCRγ (top) or TCRδ (bottom) sequences that are public per an individual’s repertoire for cCMV^−^ (blue) or cCMV^+^ (orange). Each point is an individual subject and clonotypes are defined using the amino acid sequence for the TCRδ or TCRγ chain. (D) Scatterplot showing the relationship between publicity and the number of unique nucleotide sequences encoding each amino acid clonotype, with a linear trendline and Pearson correlation coefficient and *P* value for TCRγ (left) or TCRδ (right). Total number of unique, public CDR3δ sequences = 70; total number of unique, public CDR3γ sequences = 948. For (A) and (C), statistical testing was done via Wilcoxon rank-sum test.

We compared the overall repertoire diversity by calculating the proportion of each individual’s repertoire that was composed of a unique CDR3δ or CDR3γ sequence found on only one cell. While unique sequences comprised 80% to 90% of the repertoire in uninfected neonates, among cCMV^+^ neonates, unique CDR3δ and CDR3γ sequences comprised a significantly smaller proportion of the total repertoire (Fig. 4A), indicating the presence of expanded clonotypes within these individuals. These oligoclonal expansions were further evident when examining the average cumulative proportion of the top 10 sequences for the TCRδ and TCRγ chains. The additive proportion of the repertoire was much higher for each clone in cCMV-infected individuals compared to uninfected individuals (Fig. 4B).

We also assessed the degree to which γδTCR sequences were “public,” or shared across individuals in this study. There was no difference in the overall proportion of public TCRs between cCMV-infected and uninfected infants for either TCRδ or TCRγ (Fig. 4C). However, for both TCRγ and TCRδ, there was a striking tendency for clonally expanded TCRs to also be public (Fig. 3), perhaps suggesting the existence of highly conserved TCR sequences that can bind viral or host-encoded ligands in the setting of cCMV. The publicity of TCRγ was, on average, higher than that of TCRδ, consistent with prior reports.^22^^,^^28^ Interestingly, for the TCRγ chain, there were often multiple nucleotide sequences—in some cases, more than 10—encoding the same amino acid clonotype (Fig. 4D). These multiple encoding nucleotide sequences were often a mix of public and private sequences (Fig. S5). Furthermore, this diversity of TCRγ nucleotide sequences displayed a direct relationship to the level of publicity: The more public a clonotype was, the more likely it was to have multiple nucleotide sequences encoding it. In contrast, these trends were much less pronounced for TCRδ, which showed much lower publicity overall, and less diversity in nucleotide sequences per amino acid clonotype (Fig. 4D). These findings suggest that “convergent recombination” drives the publicity of the Vγ repertoire in Vδ1^+^ cells, as has been previously postulated for Vδ2^+^Vγ9^+^ cells and the public Vβ repertoire of CD8^+^ T cells.^69^^,^^70^

### Vδ1^+^ T cells from cCMV^+^ individuals are enriched for fetal-associated TCR characteristics

Prior studies have shown that γδ T cells that arise during early fetal development share a set of distinct TCR features that distinguishes them from those that predominate in late gestation and postnatally. These features include a low number of N additions (random nucleotide additions within the gene junctions), shorter CDR3 lengths, and preferential use of particular joining (J) genes.^71^ Because cCMV infection often occurs during a window in which these early progenitors predominate, we investigated whether congenital infection results in a persistent expansion of γδ T cells from this early fetal wave and whether cells deriving from these progenitors retain distinct programming. To investigate this, we first compared the overall γδTCR repertoires of cCMV-infected and uninfected infants for the presence of features associated with γδ T cells generated during an early fetal wave.

**Figure 5.**
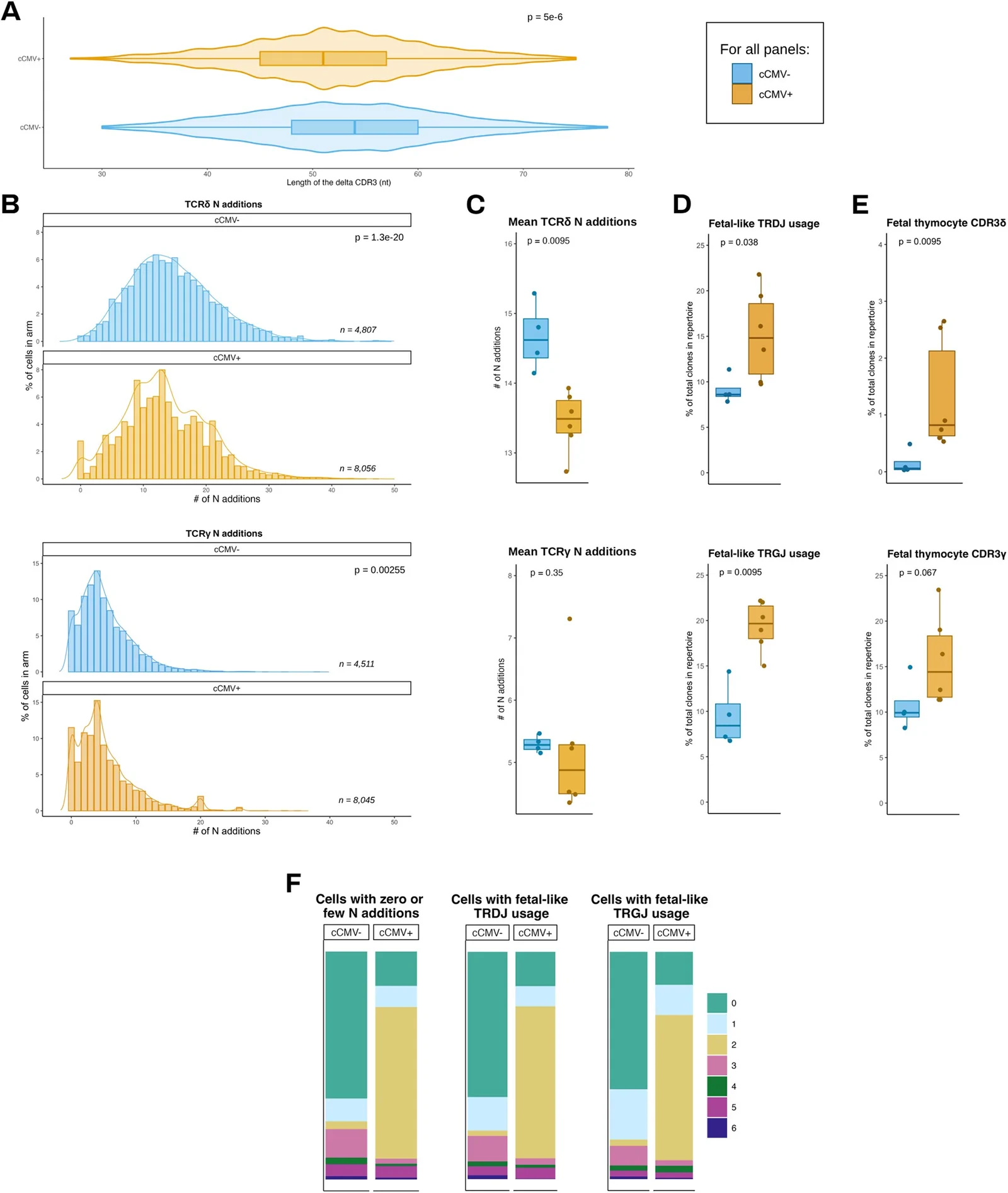
Vδ1^+^ TCRs in cCMV^+^ neonates are enriched for fetal-associated features. (A) Violin plots of the length in nucleotides of the CDR3δ for cells from cCMV^−^ or cCMV^+^ neonates. Box-and-whisker plots within show median and IQR per group. (B) Histograms with density overlay of the number of N additions in TCRδ (top) and TCRγ (bottom) for cells from cCMV^−^ or cCMV^+^ neonates. (C) Mean number of N additions per individual in TCRδ (top) and TCRγ (bottom) for cells from cCMV^−^ or cCMV^+^ neonates. (D) Percentage of cells with fetal-like *TRDJ* (top) or *TRGJ* (bottom) usage for cells from cCMV^−^ or cCMV^+^ neonates. (E) Percentage of cells with CDR3δ (top) or CDR3γ (bottom) sequences found in 24–25 gestational week fetal thymi for cells from cCMV^−^ or cCMV^+^ neonates. (F) Cluster distribution for cells with zero or few N additions (cCMV^−^  *n* = 208, cCMV^+^  *n* = 735), with fetal-like TRDJ usage (cCMV^−^  *n* = 421, cCMV^+^  *n* = 1,333), or with fetal-like TRGJ usage (cCMV^−^  *n* = 430, cCMV^+^  *n* = 1,637). For (A–E), color corresponds to cCMV status (cCMV^−^ = blue; cCMV^+^ = orange) and *P* values were determined by Wilcoxon rank-sum test. For TCRδ: cCMV^−^  *n* = 4,807, cCMV^+^  *n* = 8,065. For TCRγ: cCMV^−^  *n* = 4,551, cCMV^+^  *n* = 8,045. For (C–E), median and IQR are shown; each point is an individual.

We found that TCRs of cCMV^+^ infants had significantly shorter median CDR3δ lengths than those of cCMV-uninfected infants (Fig. 5A). In addition, the distributions of the number of N additions in the TCRγ and TCRδ were significantly left-shifted in cCMV^+^ neonates compared to uninfected neonates, indicating that there are fewer N additions within both these TCR chains (Fig. 5B). Specifically, cCMV^+^ infants were noted to have an enrichment of cells with zero TCRδ N additions (2.78% vs 0.40% in cCMV^−^). Compared to TCRδ, the overall distribution of TCRγ N additions appeared to be less impacted by cCMV; however, there was still a higher proportion of cells with zero N additions in cCMV-infected neonates (11.5% vs 8.43%) (Fig. 5B). The enrichment of cells with zero and few N additions was further confirmed by calculations of the mean number of TCRδ N additions per individual; cCMV^+^ neonates had significantly lower means compared to cCMV^−^ (Fig. 5C). Mean TCRγ N additions also trended lower in cCMV^+^ neonates (Fig. 5C).

We also analyzed *TRDJ* usage, as it has been shown that fetal thymocytes preferentially use *TRDJ2* or *TRDJ3*, whereas the vast majority of postnatal thymocytes use *TRDJ1.*^35^ We found that cCMV-infected neonates had a higher percentage of γδ T cells with fetal-like *TRDJ2* and *TRDJ3* usage (Fig. 5D). We observed a similar trend for *TRGJ* usage, with a higher frequency of cells expressing *TRGJP1* (the *TRGJ* gene preferentially used in early fetal-origin cells) in infants with cCMV (Fig. 5D).^35^ Last, we investigated whether CDR3 sequences that we observed in cCMV^+^ and cCMV^−^ infants in our cohort matched previously reported γδTCR sequences from human fetal thymocytes at mid-gestation. Using publicly available single-cell TCR repertoires from 6 fetal thymi (estimated gestational ages from 14 to 22 weeks),^42^ we observed that for both CDR3δ and CDR3γ, cCMV^+^ infants had a higher proportion of CDR3 sequences matching those present in fetal thymocytes compared to cCMV^−^ infants (Fig. 5E).

Cells with fetal-like TCR features were not only more frequent in cCMV^+^ infants, but they were also more likely to adopt a cytotoxic effector phenotype. In cCMV^+^ infants, the majority of γδ T cells with fetal-like TCR characteristics exhibit a cytotoxic effector-differentiated phenotype and appear in cluster 2 (Fig. 5F). In contrast, in uninfected infants, cells with similar fetal-like γδTCR were less frequent and predominantly maintained a naïve phenotype (cluster 0).

Examination of the TCR repertoire revealed that infection with CMV in utero results in a significant enrichment of γδ T cells with characteristics associated with early fetal development and drives differences in their transcriptional programming. This suggests that cCMV infection causes the expansion and differentiation of cells derived from early progenitors that are maintained until birth.

### Clonally expanded γδ cells in cCMV^+^ neonates have cytotoxic programming and fetal-derived TCR characteristics

**Figure 6.**
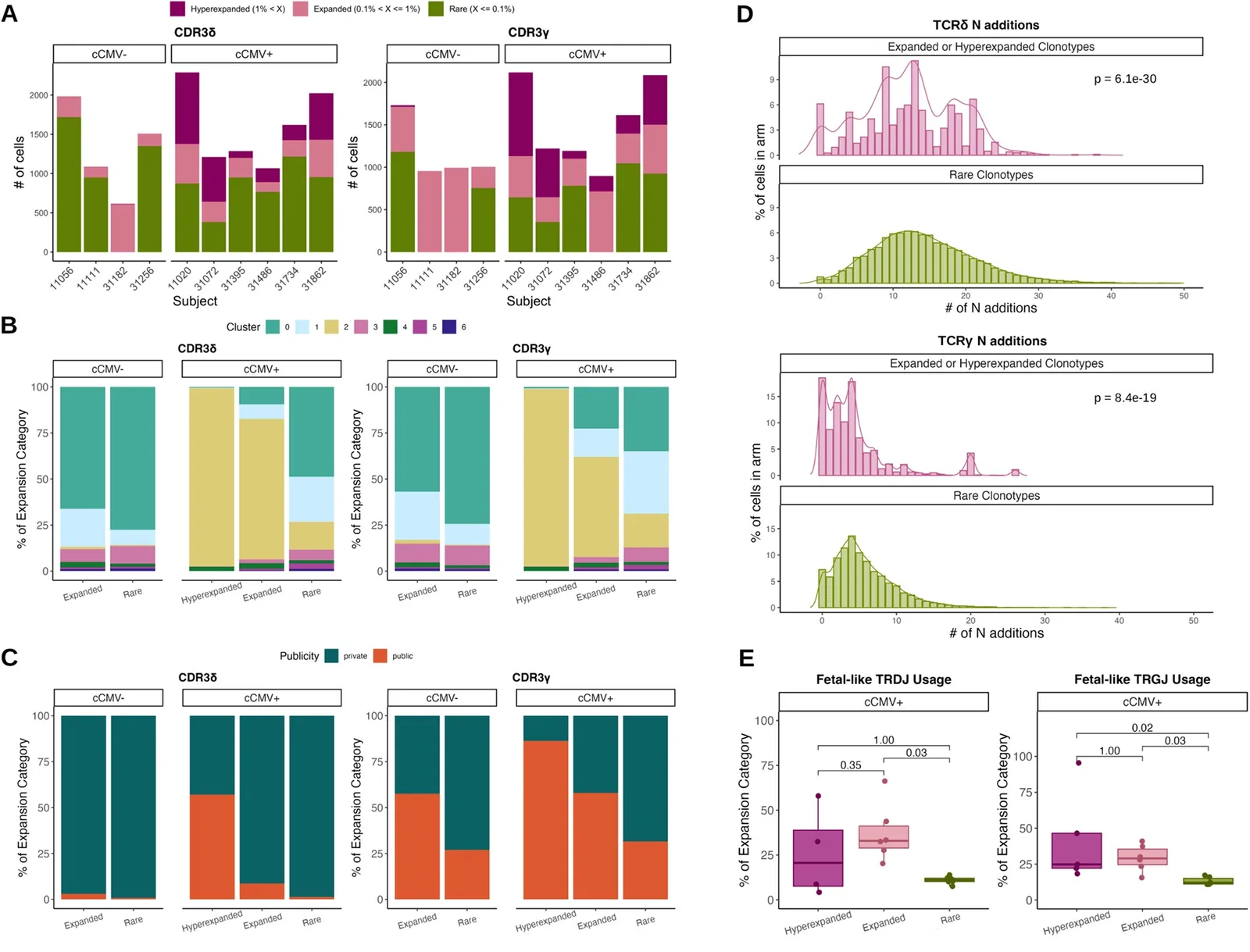
Clonally expanded Vδ1^+^ T cells in cCMV^+^ neonates have cytotoxic programming and fetal-derived TCR characteristics. (A) Number of cells in hyperexpanded (>1% of the repertoire), expanded (0.1%–1%), or rare (<0.1%) clonal expansion categories per cCMV^−^ or cCMV^+^ individual. Expansion was calculated within an individual’s repertoire. (B) Proportion of cells in each cluster per clonal expansion category for cells from cCMV^−^ or cCMV^+^ infants. (C) Proportion of cells in each clonal expansion category that are public vs private for cells from cCMV^−^ or cCMV^+^ infants. For (A–C), CDR3δ left, CDR3γ right; hyperexpanded: cCMV^+^  *n* = 2,124; expanded: cCMV^−^  *n* = 463, cCMV^+^  *n* = 1,820, rare: cCMV^−^  *n* = 6,734, cCMV^+^  *n* = 7,574. (D) Top: Histograms with density overlay of the number of N additions in TCRδ for cells in the expanded or hyperexpanded clonal expansion categories (*n* = 3,420) or the rare category (*n* = 9,031). Bottom: Histograms with density overlay of the number of N additions in TCRγ for cells in the expanded or hyperexpanded clonal expansion categories (*n* = 3,543) or the rare category (*n* = 9,053). *P* values were determined by Wilcoxon rank-sum test. (E) For cCMV^+^ individuals, the percentage of cells within each clonal expansion category with fetal-like *TRDJ* usage (left) and fetal-like *TRGJ* usage (right). Each point is an individual. *P* values were determined by a Kruskal–Wallis with Dunn post hoc test and Bonferroni correction.

Next, we focused on clonally expanded Vδ1^+^ T cells within cCMV^+^ individuals to characterize their transcriptional programming and TCR characteristics. The single-cell immune profiling package scRepertoire was used to calculate the frequency of each TCR within an individual infant’s repertoire and categorize them as rare (<0.1% of the repertoire), expanded (0.1%–1%), or hyperexpanded (>1%).^53^ Confirming what was observed from the treemap plots and computed accumulation frequencies (Figs. 3 and 4B), the repertoires of cCMV^+^ infants included a greater frequency of γδTCR clonotypes that were defined as “expanded” compared to cCMV^−^ infants. Furthermore, only cCMV^+^ individuals had “hyperexpanded” clonotypes (Fig. 6A), for both the CDR3δ and CDR3γ.

Of note, one of the expanded clones matched a previously described public fetal clone (δ1- CALGELGDDKLIF/γ8-CATWDTTGWFKIF) that was detected in nearly all of the cCMV^+^ neonates in a European study.^27^ In our cohort, this germline-encoded CDR3δ was present in 5 of 6 infants with cCMV but was absent in all 4 cCMV^−^ neonates (Table S5). Additional expanded CDR3δ clonotypes were observed, several of which were present in more than one cCMV^+^ infant, although some of these clonotypes were present in uninfected infants as well. The expanded CDR3δ clonotypes paired with a wide variety of TRVG genes (Table S5), including *TRGV9*, which is notable, as it was previously reported that expansion of γδ T cells in CMV-infected newborns is restricted to Vγ9^−^ cells.^27^ This broad Vγ chain usage suggests that many Vδ1^+^ T cells, and not just the previously reported Vγ8^+^Vδ1^+^ cells, respond to CMV infection in utero, although the reactivity of these novel clonotypes to CMV-infected cells remains to be confirmed.

Having defined these expansion categories, we sought to determine whether there was common functional programming between the expanded clones and whether this was influenced by in utero CMV exposure. We leveraged paired single-cell sequencing to assess the relationship between transcriptional programming and clonal expansion. We observed that among cCMV-uninfected neonates, most cells, whether from rare or expanded clonotypes, clustered with naïve cells (cluster 0). In contrast, among cCMV^+^ infants, clones that were expanded and hyperexpanded were predominantly in the highly cytotoxic cluster 2, whereas rare clones were more likely found in the naïve cluster 0 and cluster 1 (Fig. 6B).

In addition, cells with expanded or hyperexpanded CDR3δ were more likely to be public than rare clonotypes, specifically in cCMV-infected neonates, but not in cCMV-uninfected neonates (Fig. 6C). Interestingly, this was not the case with CDR3γ, for which expanded clonotypes were more often public, regardless of cCMV exposure. This reflects the less diverse and more public nature of CDR3γ amino acid sequences at baseline, whereas CDR3δ sequences tend to be more variable and private.

We next assessed whether the clonally expanded Vδ1 cells possessed TCR features indicative of early fetal origin. Comparison of the distributions of TCRδ or TCRγ N additions between expanded and hyperexpanded clonotypes versus rare clonotypes in cCMV^+^ individuals showed that indeed the expanded and hyperexpanded clonotypes were enriched for CDR3 sequences with few or no N additions (Fig. 6D). The expanded clones also had a bias toward usage of the fetal-like *TRDJ2* or *TRDJ3* genes as well as the fetal-like *TRGJP1* (Fig. 6E), further suggesting that they disproportionately derive from an early wave of fetal development.

In conclusion, combined scRNA-seq and scTCR-seq revealed an oligoclonal expansion of public, highly cytotoxic, effector γδ T cells in the cord blood of neonates driven by in utero infection with CMV. We additionally found an enrichment for characteristic fetal γδTCR features that suggest these expanded cells preferentially derive from early progenitors.

### Fetal-like γδ T cells display a cytotoxic, NK-like gene expression program

Finally, we sought to obtain a broader, unbiased view of the transcriptional differences between fetal-like and adult-like γδ T cells found in cord blood. In general, cells were significantly more likely to adopt a cytotoxic effector phenotype and be assigned to cluster 2 if they possessed fetal-like TCR features than those without, including having zero or few N additions (χ^2^  *P* < 2.2e-16), fetal TRDJ usage (χ^2^  *P* < 2.2e-16), and fetal TRGJ usage (χ^2^  *P* < 2.2e-16). We sought to further investigate transcriptional profiles of γδ T cells stemming from fetal origin. Based on prior studies demonstrating the distinct γδTCR characteristics of fetal thymocytes,^35^ we reasoned that cells having 0 to 4 N additions in the TCRδ were highly likely to originate from early fetal progenitors. We compared gene expression of these cells to that of cells with N additions above the median (>12), which are unlikely to arise from fetal progenitors. Unbiased clustering of γδTCRs from neonatal blood identified low/no N additions as characteristic of fetal-like γδ T clusters^62^; moreover, in explorations of our data using either the KAy-means for MIxed LArge data (KAMILA) clustering algorithm^72^ or the built-in Seurat subclustering function, the low versus high N addition dichotomy was consistently a variable that distinguished clusters. Thus, using this framework, we performed differential gene expression on fetal-like versus adult-like Vδ1^+^ T cells in our dataset using MAST. To ensure robust modeling, we included subject as a random effect to account for pseudoreplication, while cCMV status and cluster identity were included as covariates.

**Figure 7.**
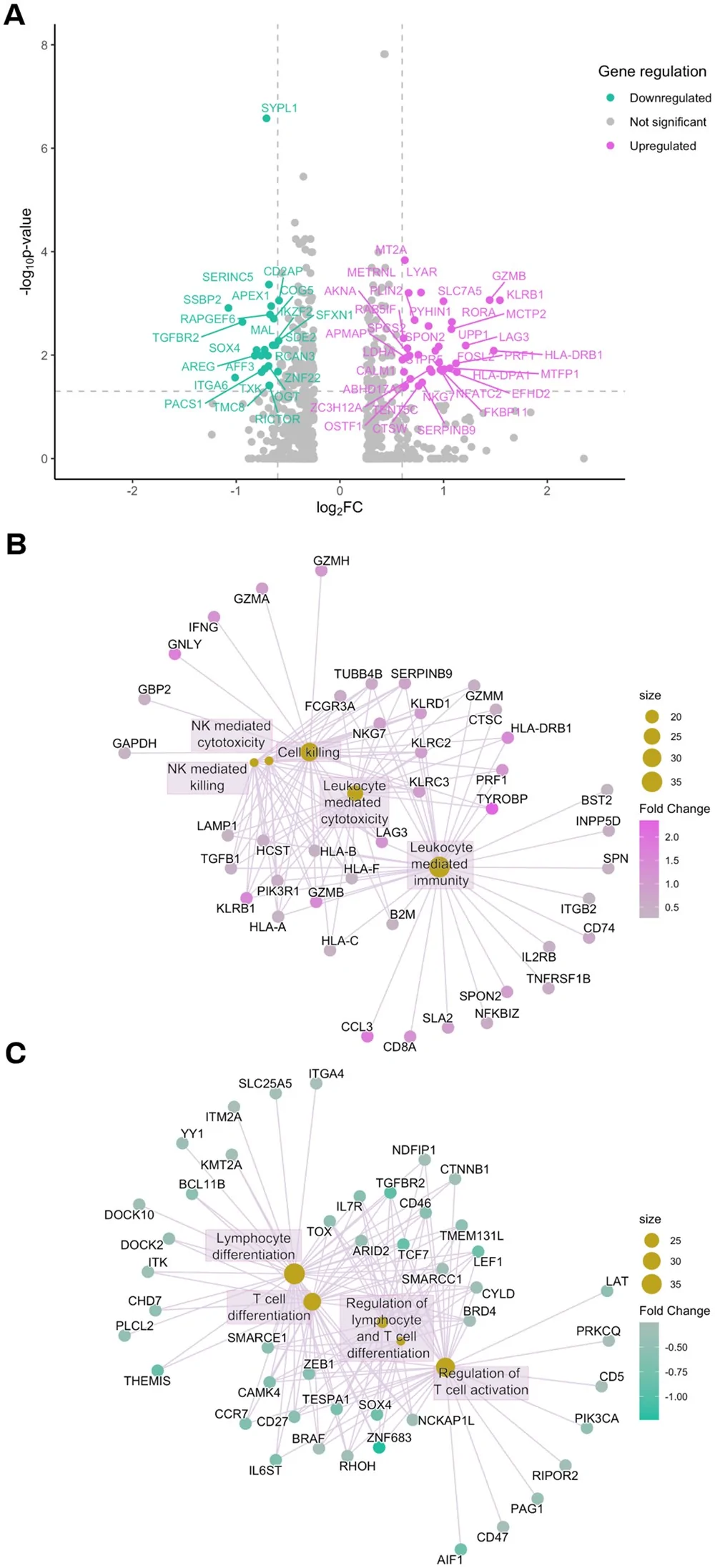
Fetal-derived Vδ1^+^ T cells display NK cell–like transcriptional programming. (A) Volcano plot depicting genes significantly upregulated (magenta) or downregulated (teal) in fetal-like Vδ1^+^ T cells vs adult-like Vδ1^+^ T cells. Horizontal dashed line: adjusted *P* = 0.05; vertical dashed lines: fold change = −1.5-fold or 1.5-fold. (B) Gene-concept network plot depicting the top 5 enriched GO terms for genes upregulated in fetal-like vs adult-like Vδ1^+^ T cells. (C) Gene-concept network plot depicting the top 5 enriched GO terms for genes downregulated in fetal-like vs adult-like Vδ1^+^ γδ T cells. For (B) and (C), nodes are the enriched GO terms and the termini the associated DEGs. Size of the node indicates how many genes were found in that GO term.

Fetal-like cells had higher expression of cytotoxic, natural killer (NK)–associated genes like *NKG7*, *GZMB*, *PRF1*, and *TYROBP*, consistent with a pre-programmed effector bias^1^^,^^27^^,^^31^^,^^35^ (Fig. 7A). In addition, we observed downregulation of *TGFBR2* and *SMC4*, both of which promote TGF-β signaling.^73^^,^^74^ TGF-β inhibits T-cell differentiation into effectors^75^^,^^76^; therefore, inhibition of this pathway would, in turn, lead to enhanced effector differentiation. GO enrichment and subsequent network analyses elucidated and confirmed the overall transcriptional programs possessed by fetal-like γδ T cells. As expected from the listed upregulated genes, enriched GO terms included “NK-mediated killing” and “NK-mediated cytotoxicity” (Fig. 7B), while downregulated terms included both those related to differentiation (lymphocyte and T-cell differentiation) and those related to regulation of function (“regulation of T-cell activation,” “antigen receptor-mediated signaling pathway”) (Fig. 7C).

In sum, unbiased differential gene expression analysis comparing cord blood γδ T cells possessing fetal-like versus adult-like TCRs demonstrated that fetal-like cells are marked by an NK-like transcriptional program that equips them for cytotoxic effector function in utero.

## Discussion

Human antiviral immune defenses evolve rapidly during fetal development and the first years of life in ways that are critically important for viral containment. As the first T cells to develop during human gestation, γδ T cells are on the front line of fetal host defense. However, few studies have examined how fetal γδ T cells respond to a perinatal pathogen. In this report, we found that CMV infection in utero drives an oligoclonal expansion of γδ T cells that adopt a highly cytotoxic effector differentiation profile and are enriched for TCR features that suggest they are derived from an early fetal progenitor population.

In agreement with prior reports,^27^^,^^18^^,^^22^ we found that the increased frequency of γδ T cells in cCMV^+^ individuals was due to oligoclonal expansion of particular Vδ1^+^ clones that are highly effector-differentiated and exhibit cytotoxic effector programming. Interestingly, and in contrast to adult CMV infection,^28^^,^^22^ the majority of these expanded clonotypes were public, or shared among multiple infants. A prior study identified a CMV-reactive public Vγ8^+^Vδ1^+^ clone that was shown via spectratyping to be present in the cord blood of 5 of the 6 cCMV^+^ infants studied, but was absent in the circulating repertoire of CMV-uninfected infants at birth.^27^ Remarkably, a subsequent analysis of fetal thymocytes noted this germline-encoded TCR sequence to be present in fetal thymocytes at mid-gestation.^35^ That expansion of this same γδTCR is seen in Ugandan infants from a distinct geographic area implies that this is a widely conserved response to CMV. In addition to this previously identified δ1-CALGELGDDKLIF/γ8-CATWDTTGWFKIF clonotype, we found numerous other clonotypes expanded in neonates with cCMV, many of them public. Given the long-term co-evolution of CMV and humans, this raises the intriguing prospect that these TCR sequences have been evolutionarily conserved to mediate antiviral CMV effector functions in utero. Further structural analysis of these γδTCRs may identify potential commonalities in structure or biochemical properties, laying the groundwork for identification of viral or host-encoded Vδ1 TCR ligands, which thus far remain largely unknown.^23^^,^^25^

Notably, we found that many of the expanded γδ T clonotypes possessed shared TCR features that are suggestive of early fetal origin, including a low number of random N additions.^35^ The TCR repertoire of fetal T cells (both αβ and γδ) is less diverse and more broadly reactive than that of adults.^77^ Elegant studies have recently shown that the low junctional diversity seen in γδ T cells that derive from early progenitor populations is due to the low activity of TdT (terminal deoxynucleotidyl transferase), the enzyme that catalyzes the random insertion of N nucleotides.^35^ Fetal hematopoietic stem and precursor cells have intrinsically high expression of the RNA-binding protein Lin28b, which suppresses TdT expression. The resulting low-diversity γδTCR repertoire may reflect the distinct priorities of host defense in early life, as both B and T cells arising during early development appear to favor broad reactivity over specificity and rapid effector action over memory formation.^77^ Intriguingly, Lin28b may act broadly as a developmental switch between the fetal and adult “layers” of the immune system, as it suppresses the *let-7* family of microRNAs, which influences the transcriptional shift from the effector-biased expression program of early life toward the generation of immune memory in later life.^35^^,^^77^

By leveraging paired transcriptomic and TCR-seq of individual γδ T cells to identify those with TCRs characteristic of fetal progenitor origin, we were able to discern differences in the global gene expression programs of γδ T cells arising from this early wave of development. Overall, we found that these early fetal-origin γδ T cells are poised for rapid effector function and possess transcriptional programs that are heavily NK-like. The resemblance of γδ T cells to NK cells resides not only in their highly cytotoxic programming and expression of cytotoxic granule–associated molecules, but other potential functions as well. In particular, we observed that γδ T cells in cCMV^+^ neonates upregulate expression of the Fc receptor CD16. We reported in a previous study that the frequency of CD56^neg^CD16^+^ NK cells is increased in neonates with cCMV and that NK-like CD16^+^CD8^+^ cells also expand following in utero CMV exposure.^78^ Thus, the developing fetus is armed with multiple populations of Fc receptor–expressing lymphocytes that can leverage transplacentally acquired maternal antibodies for antibody-dependent cellular cytotoxicity. In line with this model, recent studies have shown that high-avidity maternal CMV-specific antibodies that are able to engage FcR-mediated cellular immune responses correlate with protection against cCMV transmission.^79^^,^^80^ The ability to harness the specificity of maternal IgG via Fc-mediated immune functions may provide a critical first line of defense against viral infection at a time when the fetus lacks acquired immune memory. This collaboration with maternal-origin antibodies may be one of the many tools evolved by γδ T cells to bridge innate and adaptive immunity in the fetus.

An important limitation of our study is that the timing of mother-to-infant CMV transmission likely varied among participants and could not be determined, as vertical transmission is a clinically silent event. Because human gestation is a period of rapid immune development, the antiviral response of the fetus may differ substantially between early and late gestation. Severe sequelae of cCMV, such as microcephaly, are more likely to occur in infants infected during the first trimester,^81–84^ and currently no therapies are available to mitigate the devastating effects of fetal infection, even when it is identified early. The ability of γδ T cells to mediate rapid HLA-unrestricted antiviral effector functions raises the intriguing prospect of incorporating these broadly CMV-reactive γδTCRs into cellular therapies that could be administered to the fetus in utero. Moreover, because γδ T cells do not require HLA for antigen presentation, they are not susceptible to the CMV evasion strategy of HLA downregulation.^85^ Although we did not observe a correlation between γδ T-cell frequencies and clinical sequelae at birth, it would be intriguing to examine this relationship in a larger cohort of cCMV^+^ infants. While cCMV infection has devastating consequences for some infants, the majority remain asymptomatic,^82^^,^^84^ and it is possible that γδ T cells contribute to a partially protective immune response that enables most infants to evade sequelae. Supporting this, murine models have shown that in circumstances where conventional αβ T cells are impaired or absent, γδ T cells alone are able to directly restrict CMV replication and subsequently minimize pathology.^17^^,^^86^

In summary, we have demonstrated that γδ T cells expand in response to CMV infection in utero and exhibit a high degree of proliferation, activation, and memory differentiation. This expansion is comprised of highly cytotoxic, oligoclonally expanded cells that frequently possess public, fetal-like TCRs. These findings further our understanding of the role of γδ T cells in early life ontogeny and their development and differentiation in response to infection in utero. As rapid, broadly reactive, and innate-like effectors, γδ T cells may provide a critical defense during fetal and early life, when adaptive immune responses are lacking.

## Supplementary Material

vkag099_Supplementary_Data

## Data Availability

The flow cytometry data supporting this study will be made available in ImmPort (https://www.immport.org). In addition, the sequencing data generated and analyzed during this study will be deposited in the Gene Expression Omnibus (GEO) database (https://www.ncbi.nlm.nih.gov/geo). All other relevant data supporting the findings of this study are available from the corresponding author upon reasonable request.
